# Clinical Oncology

**Published:** 1991-12

**Authors:** V. L. Barley


### Book Reviews

**CLINICAL ONCOLOGY**
**By Gareth J. G. Rees**
Castle House Publications. 1989.
ISBN 0 71940 133 X. Price £12.95. pp. 356 + x

This book aims to give basic information on the management of adults with cancer for junior hospital doctors. The first half, covering the general principles and practice of oncology, provides an excellent introduction to the philosophy of treating patients with cancer. As expected, there are chapters on tumour biology, surgery, radiotherapy, chemotherapy and pain control. Dr. Rees gives a concise but adequate account, suitable for those not needing to specialise in oncology. Moreover he includes a comprehensive survey of associated complications such as metabolic disturbances and immunosuppression and covers also the rehabilitation and psychological needs of patients.

The second half consists of 22 chapters on the common malignancies, following a "lecture notes" style, which are informative and comprehensive. At the end of each chapter Dr. Rees lists the salient messages for a doctor looking after such patients on the ward and these "patient points" would provide useful rapid reference or reminders.

Altogether this is an excellent book for any junior doctor managing patients with cancer which can also be used as a sound basis for further oncological study.

V. L. Barley

